# Syringocystadenoma Papilliferum of the Left Gluteal Region in an Adult Female Patient: A Case Report

**DOI:** 10.7759/cureus.46220

**Published:** 2023-09-29

**Authors:** Karine Kasti, Lauren Offield, Rupa Seetharamaiah

**Affiliations:** 1 College of Medicine, Florida International University, Herbert Wertheim College of Medicine, Miami, USA; 2 Surgery, Florida International University, Herbert Wertheim College of Medicine, Miami, USA; 3 Surgery, Baptist Hospital of Miami, Miami, USA

**Keywords:** surgical management, dermatopathology, general surgery, surgical pathology, syringocystadenoma papilliferum

## Abstract

Syringocystadenoma papilliferum is a rare, benign adnexal neoplasm most often found on the scalp, head, and neck region as a solitary hairless plaque, verruca, or nodule. Lesions may arise sporadically or from a pre-existing sebaceous nevus. We report the case of a 56-year-old female who presented with an indurated multilobulated cystic mass in the left buttock region. Excisional biopsy demonstrated the histological picture of syringocystadenoma papilliferum. The interest of this case report lies in the rarity of syringocystadenoma papilliferum and its unusual presentation in the left buttock region.

## Introduction

Syringocystadenoma papilliferum is a rare benign adnexal neoplasm most seen in childhood or adolescence. Lesions may arise sporadically or from a pre-existing nevus sebaceous. It is seen most often in the scalp, head, and neck region as a solitary hairless plaque, verruca, or nodule that may be exudative. Cases have also been reported in the extremities and rarely in the groin and anogenital regions [1- 4]. However, no morphological difference between anogenital syringocystadenoma papilliferum and those in other locations has been identified [5]. Histologically, it is characteristic of ducts that connect to the surface which contain papillary processes lined by epithelial cells and apocrine or eccrine glands [6]. However, the histology of these tumors is variable in their clinical presentation and has not been well defined, but some histological markers associated with syringocystadenoma papilliferum include GATA3, CK7, BRAF V600E, and HRAS [5,7]. 

## Case presentation

We report the case of a 56-year-old female with no significant past medical history who presented with a large tri-lobulated mass to the left buttock. Pathology revealed an atypical squamous proliferation with verrucous features and indeterminate margins measuring 16 x 13 x 5 cm.

The patient first noted intermittent swelling and tenderness of the gluteal area in January 2022; however, she did not seek care until two months later when she experienced an episode of acute pain in her left buttock region. In March, the patient sought out care from an urgent care clinic and was prescribed doxycycline and topical bacitracin with no improvement of symptoms. Ten days later, the patient presented to the emergency department complaining of pain and redness to the left buttock.

**Figure 1:**
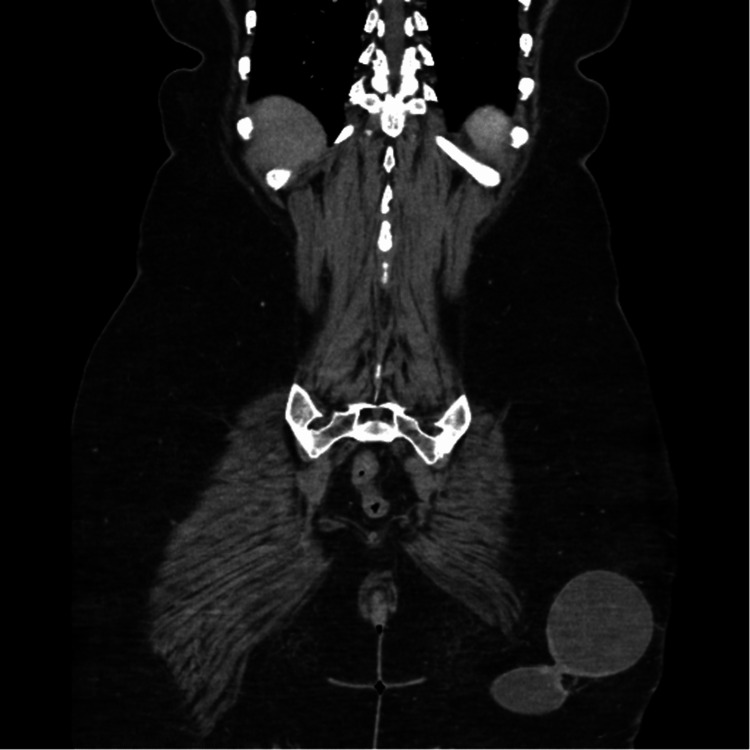
Sagittal CT scan showing a large, approximately 12.5 cm complex cystic trilobed mass in the subcutaneous left buttock soft tissues. The largest of the 3 lobes of this mass is located superiorly and laterally and measures 8.5 x 6.5 cm. A contiguous superior medial lobe measures 2.8 x 1.9 cm. A more inferior medial lobe measures 7 x 4.3 cm.

Workup in the emergency department showed no fever and normal white blood cell count. A computed tomography (CT) abdomen/pelvis with intravenous (IV) contrast demonstrated a complex cystic mass in the subcutaneous left buttock soft tissue superficial to the left gluteus musculature (Figure 1). The largest of the three lobes was located superiorly and laterally, measuring 8.5 x 6.5 cm. A contiguous superior medial lobe measured 2.8 x 1.9 cm. A more inferior medial lobe measured 7.0 x 4.2 cm. There were areas of nodular peripheral enhancement, most pronounced in the left lateral aspect of the inferior lobe. This mass extended approximately 12 cm in craniocaudal dimension. No significant adjacent fat stranding or inflammatory changes were noted. Physical exam showed a very sensitive, mobile, and superficial mass that is warm to the touch with erythema and induration. Given the size of the mass, general surgery was consulted in this case and the decision was made to resect the mass based on size and invasion of the mass for excision and pathological identification.

**Figure 2:**
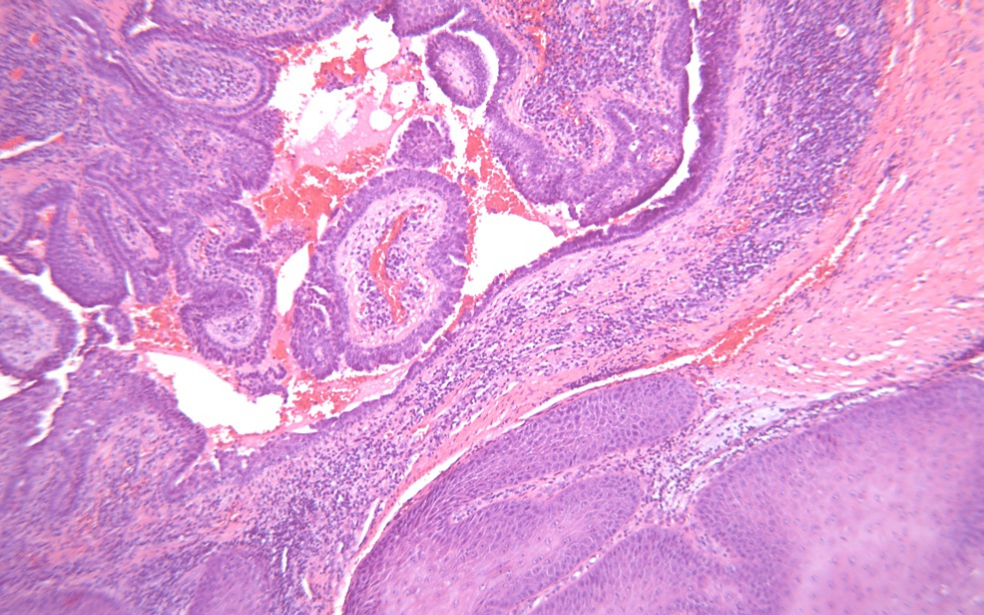
A papillary proliferation consistent with syringocystadenoma papilliferum (upper left) adjacent to a well-differentiated squamous proliferation consistent with squamous cell carcinoma (lower right).

**Figure 3:**
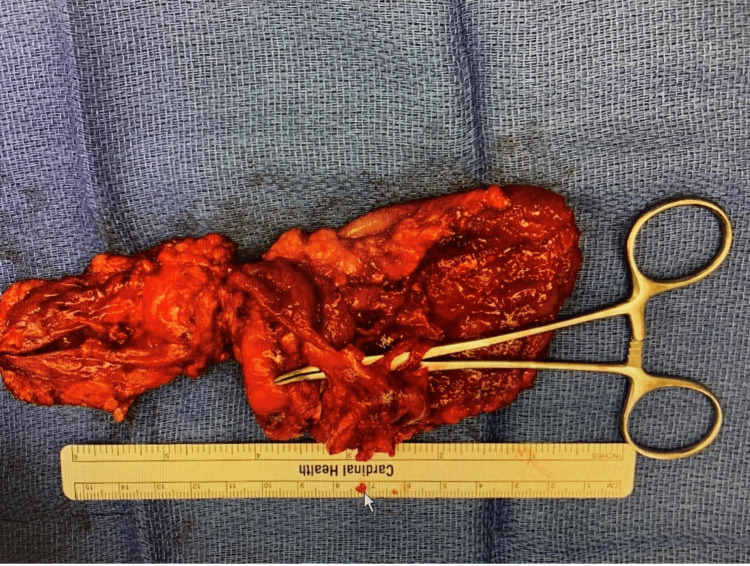
Gross image showing excised mass measured at 16 x 13 x 5 cm.

Preoperatively, the patient was given IV fluids and IV antibiotics while awaiting incision and drainage with possible excision of left buttock soft tissue to treat the overlying infection prior to surgical excision. A complex cystic trilobed mass was excised. Postoperatively, the pathology report showed an atypical squamous proliferation consistent with well-differentiated squamous cell carcinoma with verrucous features and indeterminate margins, arising in association with syringocystadenoma papilliferum (Figure 2). The gross description included a mass consisting of 16 x 13 x 5 cm in aggregate of pale tan skin, fibrofatty soft tissue, and a disrupted cyst wall (Figure 3).

Given the pathology, re-excision of the left buttock wound to assess for the margins was indicated. Re-excision pathology reported benign skin with evidence of recent manipulation including ulceration, inflamed granulation tissue, fibrosis, and negative findings for residual squamous cell carcinoma.

## Discussion

**Figure 4:**
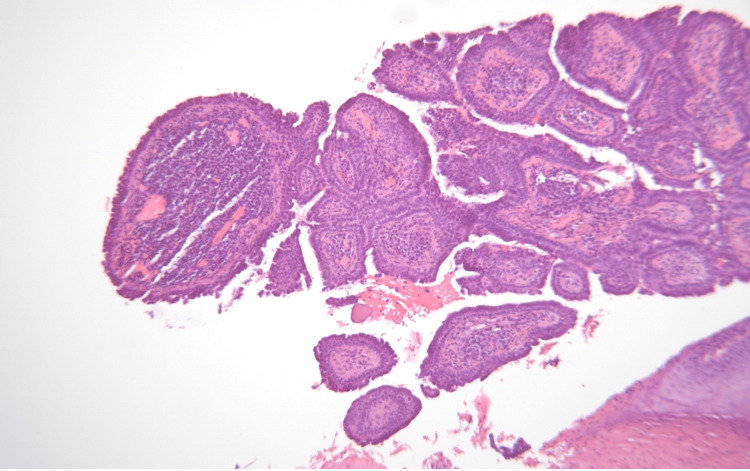
Syringocystadenoma papilliferum. The papillary lesion is lined by a double layer of small cuboidal cells.

Syringocystadenoma papilliferum is a rare hamartomatous tumor arising from pluripotent cells of apocrine or eccrine glands [8]. This case report presents an uncommon tumor in a rare location in an adult patient, one of the few case reports to be reported in the scientific literature of this presentation of the tumor. Some of the differential diagnoses that were considered include neoplasms such as myxomatous sarcoma, infection, evolving hematoma, and complicated epidermal inclusion cyst. We confirmed the diagnosis of syringocystadenoma papilliferum after a biopsy was sent to pathology postoperatively (Figure 4). The standard of treatment for syringocystadenoma papilliferum is surgical excision. The use of a carbon dioxide laser is rare but may be effective in removing tumors on anatomical sites that are not candidates for surgical excision [4]. 

In Figure 2, the finding of syringocystadenoma papillferum was seen along with squamous proliferation that was consistent with squamous cell carcinoma. Rarely, small growths occur alongside the tumor, termed contiguous squamous proliferations, which may be benign or malignant. The de novo lesions are more likely to demonstrate contiguous squamous proliferations when compared to those arising from nevus sebaceous [7]. Benign spread is more likely to have originated from adjacent skin lining, whereas the malignant spread is more likely to arise from the syringocystadenoma papilliferum itself termed the adenomatous component [7]. However, the relationship between syringocystadenoma papillferum and squamous cell carcinoma is not yet well understood, thus, it is important to highlight this case to help further the literature and provide opportunities for the pathology to be further investigated.

**Figure 5:**
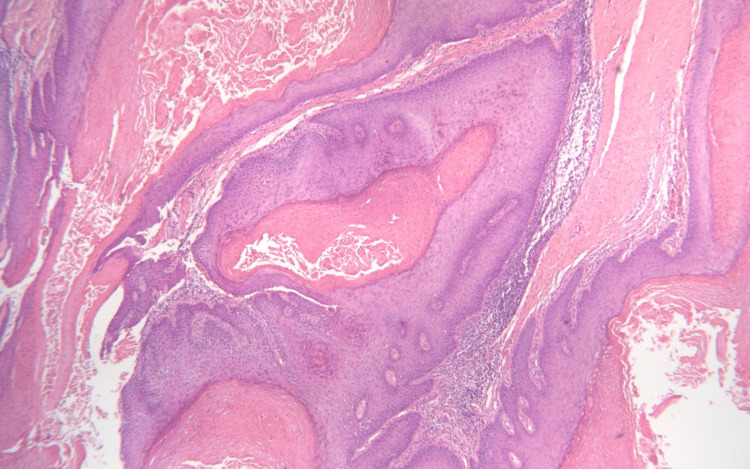
A well-differentiated squamous proliferation with focal atypia and broad fronts of invasion.

Additionally, the findings from this patient’s pathology indicated a highly hyperplastic epidermis, which could indicate reactivity to syringocystadenoma papilliferum, resembling pseudoepitheliomatous hyperplasia (Figure 5). The pathology also showed verrucous epidermis, which is often seen in cases of epidermal nevus associated with syringocystadenoma papiliferrum (Figure 5). However, no clinical signs were evident in this patient to confirm the association observed.

In 2019, Konstantinova et al. performed a report on 16 cases which provided insight into sporadic syringocystadenoma papilliferum located in the buttock and anogenital area, highlighting various morphological changes that can occur in these lesions. Some tumors showed features resembling warts, but the presence of HPV was not consistently linked to these changes [5]. In comparison to our case, they reported squamous epithelial metaplasia was the most frequently observed type of metaplasia alongside syringocystadenoma papilliferum. In one case, there was a slight atypia in the squamous epithelium, with some areas showing basaloid cells and numerous mitotic figures, creating a resemblance to undifferentiated vulvar intraepithelial neoplasia [5]. Aggarwal et al. reviewed 14 cases in 2023 and reported a majority of the syringocystadenoma papilliferum showed squamous proliferation, including four instances of verrucous hyperplasia, six cases with papillomatosis, two cases with mild squamous hyperplasia, and one case each of Bowen's disease and squamous cell carcinoma [7]. Thus, the findings of our case support what is currently found in the literature and also further aim to investigate the association between syringocystadenoma papilliferum and squamous cell carcinoma.

## Conclusions

The interest of this case report lies in the rarity of syringocystadenoma papilliferum and its unusual presentation in the left buttock region with multilobulations. This case sheds light on a rare surgical pathology finding, demonstrating an atypical squamous proliferation consistent with well-differentiated squamous cell carcinoma with verrucous features. Although benign, it is important to distinguish syringocystadenoma papilliferum from other skin tumors to ensure accurate diagnosis and treatment. The overall risk of malignant contiguous squamous proliferations to appear in syringocystadenoma papilliferum is rare. We hope this provides guidance in the future for patients with syringocystadenoma papilliferum who require surgical excision.

## References

[1] Skelton HG 3rd, Smith KJ, Young D, Lupton GP (1994). Condyloma acuminatum associated with syringocystadenoma papilliferum. Am J Dermatopathol.

[2] Steshenko O, Chandrasekaran N, Lawton F (2014). Syringocysadenoma papilliferum of the vulva: a rarity in gynaecology. BMJ Case Rep.

[3] Dufrechou L, Acosta A, Beltramo P, Pomies V, Caruso R, Salmenton GM, Alvarez M (2013). Syringocystadenoma papilliferum arising on the scrotum. Pediatr Dermatol.

[4] Pahwa P, Kaushal S, Gupta S, Khaitan BK, Sharma VK, Sethuraman G (2011). Linear syringocystadenoma papilliferum: an unusual location. Pediatr Dermatol.

[5] Konstantinova AM, Kyrpychova L, Nemcova J (2019). Syringocystadenoma papilliferum of the anogenital area and buttocks: a report of 16 cases, including human papillomavirus analysis and HRAS and BRAF V600 mutation studies. Am J Dermatopathol.

[6] Ghosh SK, Bandyopadhyay D, Chatterjee G, Bar C (2009). Syringocystadenoma papilliferum: an unusual presentation. Pediatr Dermatol.

[7] Aggarwal D, Chatterjee D, Keshavamurthy V (2023). Contiguous squamous proliferations in syringocystadenoma papilliferum: a retrospective study of 14 cases. Indian J Dermatol Venereol Leprol.

[8] Yamamoto O, Doi Y, Hamada T, Hisaoka M, Sasaguri Y (2002). An immunohistochemical and ultrastructural study of syringocystadenoma papilliferum. Br J Dermatol.

